# The biological knowledge discovery by PCCF measure and PCA-F projection

**DOI:** 10.1371/journal.pone.0175104

**Published:** 2017-04-11

**Authors:** Xingang Jia, Guanqun Zhu, Qiuhong Han, Zuhong Lu

**Affiliations:** 1 Department of Mathematics, Southeast University, Nanjing 210096, PR China; 2 State Key Laboratory of Bioelectronics, School of Biological Science and Medical Engineering, Southeast University, Nanjing 210096, PR China; 3 Department of chemistry, Nanjing Agricultural University, Nanjing 210000, PR China; 4 Department of Mathematics, Nanjing Forestry University, Nanjing 210037, PR China; University of Arizona, UNITED STATES

## Abstract

In the process of biological knowledge discovery, PCA is commonly used to complement the clustering analysis, but PCA typically gives the poor visualizations for most gene expression data sets. Here, we propose a PCCF measure, and use PCA-F to display clusters of PCCF, where PCCF and PCA-F are modeled from the modified cumulative probabilities of genes. From the analysis of simulated and experimental data sets, we demonstrate that PCCF is more appropriate and reliable for analyzing gene expression data compared to other commonly used distances or similarity measures, and PCA-F is a good visualization technique for identifying clusters of PCCF, where we aim at such data sets that the expression values of genes are collected at different time points.

## Introduction

In the process of biological knowledge discovery, the clustering and visualizing analysis plays central roles [1–3]. The clustering algorithms are used to search for patterns that provide additional insight into the biological function and relevance of genes [4, 5]. Among the most popular are unsupervised clustering algorithms, such as K-means [5]. K-means analysis depends on choosing an appropriate distance or similarity measure that takes into account the underlying biology and the nature of the data [6]. Commonly used measures include PCC(the Pearson correlation coefficient) and Euclidean distance [7]. However, K-means can not reveal underlying global patterns in the data, or relationships between the clusters found. To complement K-means, PCA is a commonly used method for this purpose. But for most gene expression data, PCA typically gives a poor visualization [8, 9]. Because of these limitations, nonlinear dimension reduction methods have been developed that attempt to preserve local structure in the data, such as t-SNE(t-statistic Stochastic Neighbor Embedding) [8, 10, 11]. For t-SNE, it has been successful in displaying clusters of Euclidean distance [8], but it gives the poor visualizations for clusters of PCC usually.

Here, we use PCCF to measure similarity of genes, and PCA-F to display clusters of PCCF, where PCCF is the correlation coefficient of F-points, PCA-F is the principal component analysis of F-points, and F-point of a gene is constructed by the modified cumulative probability of the positively and reversely normalized gene. To evaluate PCCF measure, we apply it to group four gene expression data sets. These clustering results clearly demonstrate the statistical reliability and biological relevance of PCCF far more than other commonly used distances or similarity measures. For PCA-F, the cumulative variance of its principal components are greater than 85% for any reference data set in this paper, and far more than PCA of the normalized points. Furthermore, we demonstrate that PCA-F is able to project similar F-points in the same regions, to accurately depict distant F-points, and to accurately reveal the relationships of clusters of PCCF. These superior performances of PCCF and PCA-F benefit from the validity of F-points. The most prominent feature of F-points is that their curve shapes are almost like capital *N*. That is, F-points weaken the curve shape difference of the similar expression behavior genes. Moreover, F-points enlarge the element discrepancy of dissimilar genes by their two cumulative probability.

However, for PCA-F maps of many expression data sets, projections in their internal regions are crowded usually, where these crowded projections come from these genes that their elements are relatively equivalent. For a 2D projecting map, it needs to help an investigator in the interpretation of any particular region of the visualization, but the crowded regions can give inconvenience for the investigator. To clearly distinguish any projecting region, we propose PCA-FO that is the similarity transformation of PCA-F. For gene points, the position relationship of their PCA-FO projections is the same as their PCA-F projections, but the spaces of PCA-FO projections are more uniform compared to PCA-F.

In this study, these data sets from published studies are used to investigate and illustrate the performance of PCCF and PCA-F, including the yeast metabolic cycle data [12], K562 cell line data [13], human embryo data [14], and mouse retinal data [7]. Here, PCCF is firstly applied to divide these data sets into clusters, and then these clustering results are overlayed onto PCA-F maps. Results show that PCCF is able to group the similar expression behavior genes into the same clusters, and PCA-F is able to project genes of the same clusters together. That is, PCCF and PCA-F can be used in conjunction to understand the logic of cluster partitions and to identify co-regulated genes. We suggest that PCCF and PCA-F provide new insights for analyzing large-scale transcriptome data.

## Materials and methods

### Data set 1

The simulation data set contained 1500 four-dimensional points. These 1500 points belonged to 14 populations, and each population was constructed by four independent normal distributions, where the used normal distributions were N(10,1) and N(20,2). Obviously, N(10,1) and N(20,2) would construct 16 four-dimensional normal populations. Here, (N(10,1),N(10,1),N(10,1),N(10,1)) and (N(10,1),N(20,2),N(20,2),N(20,2)) were abandoned, (N(20,2),N(20,2),N(20,2),N(10,1)) consisted of 200 points, and each of other populations consisted of 100 points.

For points of (N(20,2),N(20,2),N(20,2),N(20,2)), all their elements were equivalent. And for points of other groups, half of their elements were relatively equivalent at least.

### Data set 2

#### NCBI GEO accession number GSE 12736

Time course microarray data was obtained at seven independent time points. Duplicate experiments were performed for each time point. Selecting genes with significant detection p-value produced 14000 probes out of total 23920 probes. Quantile normalization was carried out for each dataset at seven time points using the average expression value. It was reasoned that significant genes should show over two-fold induction at least at one time point with respect to the control sample(t = 0; before PMA treatment), and 1779 probes satisfying this requirement have been determined [13, 15].

### Data set 3

#### Yeast metabolic cycle data: NCBI GEO accession number GSE3431

This data set described the transcriptional changes in the metabolic cycle of budding yeast Saccharomyces cerevisiae [12, 14]. In this experiment, gene expression behaved in a periodic manner, comprising a non-respiratory phase followed by a respiratory phase. The transcriptome was assayed every 25 min over three consecutive cycles, resulting in 36 samples (T1-T36). These were profiled using Affymetrix YG_S98 oligonucleotide arrays. Probes that had at least three ‘present’ called as generated by Affymetrix Gene Chip software were classified as expressed and the data normalized using GeneSpring v7 per-chip normalization. Using a periodicity algorithm described in the original paper, the authors classified 3552 genes as periodic, corresponding to 3656 probe sets. From these 3552 genes, 2913 genes, expression values had greater than 5 in at least one of 36 samples selected.

### Data set 4

#### Human embryo data: NCBI GEO accession number GSE18887

The resulting matrix contained expression measurements for 5441 transcripts across 18 samples, denoted as the human organogenesis expression matrix [14] (Carnegie stages 9-14, S9-S14). A total of 5441 probe sets were identified as differentially expressed using Extraction of Differential Gene Expression (EDGE)-based methodology. Initially, Hai Fang had used SOM-SVD to identify co-expressed genes of Human embryo Data [10, 14], which identified six clusters. From their analysis, they extracted 2148 differentially expressed probe sets. We used this set of 2148 probe sets for our analysis.

### Data set 5

The raw mouse retinal data consisted of 10 SAGE libraries (38818 unique tags with tag counts ≥ 2) from developing retina taken at 2-day intervals. The samples ranged from embryonic, to postnatal, and to adult. Among the 38818 tags, 1467 tags that had counts greater than or equal to 20 in at least one of the 10 libraries were selected [7]. The purpose of this selection was to exclude the genes with uniform low expression. The counts of each tag in a SAGE library was Poisson distributed. These Poisson distributions were independent of each other across different tags and libraries [7].

### Methods

The gene expression points can be represented by the *n*-tuple of vectors, where *X*_*i*_ = {*x*_*i*1_, *x*_*i*2_,⋯, *x*_*in*_} represents the *i*-th gene, and *x*_*ij*_ represents the expression level of the *j*-th time points.

### F-points

1. *X*_*i*_ is normalized into *W*_*i*_, where
  $$\begin{array}{c} W_{i}=\left\{w_{i1},w_{i2},\cdots ,w_{in}\right\},\;\;w_{it}=\frac{x_{it}-\min\left(\min_{1\leq t\leq n}\left(x_{it}\right),0\right)}{\sum_{l=1}^{n}\left(x_{il}-\min\left(\min_{1\leq t\leq n}\left(x_{it}\right),0\right)\right)},\;t=1,2,\cdots ,n. \end{array}$$ (1)
  For genes, their expression levels may be negative at some time points, such as genes of data set 2. Here, *x*_*it*_ is substituted by $x_{it}-\min\left(\min_{1\leq t\leq n}\left(x_{it}\right),0\right)$. In fact, if all expression levels of *X*_*i*_ are nonnegative, $x_{it}-\min\left(\min_{1\leq t\leq n}\left(x_{it}\right),0\right)$ is the same as *x*_*it*_.
2. $W_{i}^{{}^{\prime }}$ is constructed, where $W_{i}^{{}^{\prime }}$ is the modified cumulative probability of *W*_*i*_, $W_{i}^{{}^{\prime }}$ is named as P-point of *X*_*i*_, and
  $$\begin{array}{c} \;\;\;\;\;\;W_{i}^{{}^{\prime }}=\left\{w_{i1}/2,w_{i1}+w_{i2}/2,w_{i1}+w_{i2}+w_{i3}/2,\cdots ,\sum {}_{s=1}^{n-1}w_{is}+w_{in}/2\right\}. \end{array}$$
3. *V*_*i*_ and $V_{i}^{{}^{\prime }}$ are constructed by *W*_*i*_, where *V*_*i*_ is the ON-point of *X*_*i*_, $V_{i}^{{}^{\prime }}$ is the modified cumulative probability of *Y*_*i*_, and
  $$\left\{\begin{array}{cc} V_{i} & =\{w_{in},w_{i(n-1)},\cdots ,w_{i2},w_{i1}\},\\ V_{i}^{{}^{\prime }} & =\{w_{in}/2,w_{in}+w_{i(n-1)}/2,\cdots ,\sum {}_{s=2}^{n}w_{is}+w_{i1}/2\}. \end{array}\right.$$
4. $W_{i}^{{}^{\prime }}$ and $Y_{i}^{{}^{\prime }}$ are merged into *F*_*i*_, where *F*_*i*_ is named as F-point of *X*_*i*_, and
  $$\begin{array}{lll} F_{i} & = & \{w_{i1}/2,w_{i1}+w_{i2}/2,w_{i1}+w_{i2}+w_{i3}/2,\cdots ,\sum_{s=1}^{n-1}w_{is}+w_{in}/2,w_{in}/2,\\ & & w_{in}+w_{i\left(n-1\right)}/2,w_{in}+w_{i\left(n-1\right)}+w_{i\left(n-2\right)}/2,\cdots ,\sum_{s=2}^{n}w_{is}+w_{i1}/2\}. \end{array}$$ (2)
  For *F*_*i*_, it is a 2*n*-dimensional vector, and the sum of its elements is *n*.

For *W*_*i*_, the last element of its cumulative probability is 1, it may lose part information of *w*_*in*_, so we select the modified cumulative probability. Since the elements of $W_{i}^{{}^{\prime }}$ and $Y_{i}^{{}^{\prime }}$ are the monotonous unabated, and

$$\begin{array}{c} \sum {}_{s=1}^{n-1}w_{is}+w_{in}/2\geq 0.5\geq w_{in}/2, \end{array}$$

the curve shape of *F*_*i*_ is almost like capital *N*. That is, F-points weaken the curve shape difference of the similar expression behavior genes. Without doubt, the curve shapes of the dissimilar expression behavior genes are similar also. However, F-points enlarge the element discrepancy of dissimilar genes by their two modified cumulative probability. That is, the curve shapes of dissimilar expression behavior genes are different *N*.

### PCCF measure

Here, PCC between *F*_*i*_ and *F*_*j*_(or $W_{i}^{{}^{\prime }}$ and $W_{j}^{{}^{\prime }}$) is defined as PCCF(or PCCP) of *X*_*i*_ and *X*_*j*_. Moreover, Euclidean distance between *F*_*i*_ and *F*_*j*_(or $W_{i}^{{}^{\prime }}$ and $W_{j}^{{}^{\prime }}$) is defined as EuF(or EuP) of *X*_*i*_ and *X*_*j*_ also.

In fact, $W_{i}^{{}^{\prime }}$ and *F*_*i*_ is able to describe as

$$\left\{\begin{array}{cc} W_{i}^{{}^{\prime }} & =\{w_{i1}^{{}^{\prime }},w_{i2}^{{}^{\prime }},\cdots ,w_{in}^{{}^{\prime }}\},\\ F_{i} & =\{w_{i1}^{{}^{\prime }},w_{i2}^{{}^{\prime }},\cdots ,w_{in}^{{}^{\prime }},1-w_{in}^{{}^{\prime }},1-w_{i(n-1)}^{{}^{\prime }}\cdots ,1-w_{i2}^{{}^{\prime }},1-w_{i1}^{{}^{\prime }}\}. \end{array}\right.$$ (3)

Based on Eq (3), EuF and EuP between *X*_*i*_ and *X*_*j*_ satisfy

$$\begin{array}{c} EuF\left(i,j\right)=\sqrt{2}EuP\left(i,j\right). \end{array}$$

That is, EuF and EuP are the same distance in essence.

But for PCCP and PCCF of *X*_*i*_ and *X*_*j*_, they are

$$\left\{\begin{array}{cc} PCCP(i,j) & =\frac{\sum {}_{s=1}^{n}\left(w_{is}^{{}^{\prime }}-\frac{1}{n}\sum {}_{t=1}^{n}w_{it}^{{}^{\prime }}\right)\left(w_{js}^{{}^{\prime }}-\frac{1}{n}\sum {}_{t=1}^{n}w_{jt}^{{}^{\prime }}\right)}{\sqrt{\left(\sum {}_{s=1}^{n}{\left(w_{is}^{{}^{\prime }}-\frac{1}{n}\sum {}_{t=1}^{n}w_{it}^{{}^{\prime }}\right)}^{2}\right)\left(\sum {}_{s=1}^{n}{\left(w_{js}^{{}^{\prime }}-\frac{1}{n}\sum {}_{t=1}^{n}w_{jt}^{{}^{\prime }}\right)}^{2}\right)}},\\ PCCF(i,j) & =\frac{\sum {}_{s=1}^{n}\left(w_{is}^{{}^{\prime }}-0.5\right)\left(w_{js}^{{}^{\prime }}-0.5\right)}{\sqrt{{(\sum {}_{s=1}^{n}\left(w_{is}^{{}^{\prime }}-0.5\right)}^{2}){(\sum {}_{s=1}^{n}\left(w_{js}^{{}^{\prime }}-0.5\right)}^{2})}}, \end{array}\right.$$ (4)

where the mean of *F*_*i*_ is 0.5. Since the means of $W_{i}^{{}^{\prime }}$ and $W_{j}^{{}^{\prime }}$ are not likely 0.5 at the same time, PCCP and PCCF of *X*_*i*_ and *X*_*j*_ have significant difference.

### PCA-F and PCA-FO

Here, (*f*_*i*_(1), *f*_*i*_(2)) is called as PCA-F projection of *X*_*i*_, where *f*_*i*_(1) and *f*_*i*_(2) are the first and second principal components of *F*_*i*_, respectively. Moreover, (*F*_*i*_(1), *F*_*i*_(2)) is extracted as PCA-FO projection of *X*_*i*_, where

$$\left\{\begin{array}{cc} F_{i}\left(1\right) & =\frac{f_{i}\left(1\right)}{\max_{1\leq j\leq m}f_{j}\left(1\right)}+\frac{n(f_{i}\left(1\right))}{m},\\ F_{i}\left(2\right) & =\frac{f_{i}\left(2\right)}{\max_{1\leq j\leq m}f_{j}\left(2\right)}+\frac{n(f_{i}\left(2\right))}{m}, \end{array}\right.$$ (5)

*m* is gene number of data set, *n*(*f*_*i*_(1)) and *n*(*f*_*i*_(2)) are the ordering number of *f*_*i*_(1) and *f*_*i*_(2), respectively. That is, all *f*_*i*_(1)(or *f*_*i*_(2)) are irstly ordered from the smallest value to the largest one, then *n*(*f*_*i*_(1))(or *n*(*f*_*i*_(2))) is obtained by the ordering number of *f*_*i*_(1)(or *f*_*i*_(2)). For instance, if *f*_*i*_(1) is the *u*-th smallest value in all *f*_*j*_(1), *n*(*f*_*i*_(1)) is *u*.

### *S*-value

The average silhouette value is a quantitative way to compare different clustering solutions [16]. For a data set, we use the average silhouette value to quantify clustering results of its normalized points, P-points and F-points. Here, we use *S*1-value to denote the average silhouette value of the data set, where

$$S1\;=\;\frac{1}{m}\;\sum_{i=1}^{m}\frac{\left(b_{i}\;-\;a_{i}\right)}{\text{max}(a_{i},\;b_{i})},$$

*a*_*i*_ is the average distance from *Y*_*i*_ to the other points in the same cluster as *Y*_*i*_, *b*_*i*_ is the minimum average distance from *Y*_*i*_ to points in a different cluster, minimized over clusters, *Y*_*i*_ is the *i*-point of a data set, and *m* is gene number of the data [16].

Moreover, we use *S*2-value to evaluate the projections in the same regions whether that come from similar points, Here, projections are firstly divided into clusters by Euclidean distance, then the cluster membership of *Y*_*i*_ is *k* if its projection belongs to the *k*-th cluster. And then, *S*2-value is obtained by the average silhouette value of *Y*_*i*_. Here, when we use *S*2-value to evaluate the quality of projections, this *S*2-value is abbreviated as *S*2-value of PCCF if the similarity of genes is defined by PCCF measure, and so on.

### *D*-plot

For a dimension reduction technique, we term it as a ‘locally valid’(or ‘globally valid’) visualization if it satisfies that the *i*-th closest neighbour(or farthest point) of a point is its *j*-th closest neighbour(or farthest point) in 2D space, and *i*, *j* and |*i* − *j*| are the relative small number, where point neighbours are located by PCC measure, while projection neighbours are located by Euclidean distance.

The local and global validity can be respectively quantified by *D*_1_-plot and *D*_2_-plot, where

$$\left\{\begin{array}{cc} D_{1}\left(b\right) & =\frac{\sum_{i=1}^{m}\sum_{a=1}^{b}\rho_{2}\left(i,a\right)}{\sum_{i=1}^{m}\sum_{c=1}^{b}\rho_{n}\left(i,c\right)},b=2,3,\cdots ,k,1\leq a,c\leq b,\\ D_{2}\left(b\right) & =\frac{\sum_{i=1}^{m}\sum_{e=1}^{b}\rho_{2}\left(i,e\right)}{\sum_{i=1}^{m}\sum_{f=1}^{b}\rho_{n}\left(i,f\right)},b=2,3,\cdots ,k,1\leq e,f\leq b, \end{array}\right.$$ (6)

*m* is point number of the data, *k* is a certain limit of local validity, *ρ*_2_(*i*, *a*) is PCC between *X*_*i*_ and its *a*-th closest neighbor in 2D space, *ρ*_*n*_(*i*, *c*) is PCC between *X*_*i*_ and its *c*-th closest neighbor in high dimensional space, *ρ*_2_(*i*, *e*) is PCC between *X*_*i*_ and its *e*-th farthest points in 2D space, *ρ*_*n*_(*i*, *f*) is PCC between *X*_*i*_ and its *f*-th farthest points in high dimensional space.

In general, when we use PCC to locate point neighbours, the closest neighbors of projections do not necessarily come from real point neighbors. That is, for the *c*-th closest neighbor of *X*_*i*_ in high dimensional space, if its projection is the *s*(*s* > *k*)-th closest neighbor of the projection *X*_*i*_, *ρ*_*n*_(*i*, *c*) does not appear in $\sum_{a=1}^{b}\rho_{2}\left(i,a\right)$. Thus,

$$\sum_{a=1}^{b}\rho_{2}\left(i,a\right)\leq \sum_{c=1}^{b}\rho_{n}\left(i,c\right),$$

Moreover, for a large scale gene expression data and a relative small *k*, *ρ*_*n*_(*i*, *c*) is usually nonnegative. Thus,

$$D_{1}\left(b\right)\leq 1,\;b=2,3,\cdots ,k.$$

Here, we connect these (*b*, *D*_1_(*b*)) into a broken line, and the broken line is named as *D*_1_-plot. Obviously, *D*_1_-plot is more close *Y* = 1, the more high dimension nearest neighbours are located close to one another in 2D maps. Similarly, *D*_2_-plot is defined, and it is more close *Y* = 1, the relationship of distant points is depicted as more accurately.

## Results

Here, all clustering results were generated from K-means with the normalized points, and PCCF, PCC, PCCP, EuF, Euclidean distance, TransChisq and PoissonC were chosen as distance or similarity measure of genes. Moreover, the number of clusters mainly came from the corresponding references. In details, Limb JK et al had divided data set 2 into 8 clusters by Euclidean [13]; Natascha B et al had divided data set 3 into 3 clusters, and data set 4 into 6 and 10 clusters by Euclidean [8]; and data set 5 had been grouped into 30 clusters by TransChisq and PoissonC measure [7, 17], respectively. Furthermore, for any clustering result, K-means iterated 1000 times at least.

### The statistical reliability of PCCF

Here, we used *S*1-value to demonstrate the statistical reliability of clusters of PCCF. For comparison, the normalized genes of each experimental data set were divided into clusters by Euclidean, PCC, PCCP, EuF and PCCF, simultaneously. For these clustering results, their *S*1-values were summarized in Table 1. For *S*1-value of clustering results within the same data, Table 1 showed that clusters of PCCF was the largest, and far more than other measures. That is, clusters of PCCF were better separated than other measures.

**Table 1:** The *S*1-values of Eu, PCC, PCCP, EuF and PCCF.

Data	Clustering number	Euclidean	PCC	PCCP	EuF	PCCF
2	8	0.24431	0.35390	0.53346	0.35242	0.55740
2	12	0.24431	0.40716	0.45752	0.32682	0.51160
3	3	0.26024	0.47487	0.41759	0.35794	0.54141
3	7	0.21686	0.29587	0.32646	0.25695	0.42808
4	6	0.36134	0.36943	0.57720	0.47554	0.70448
4	10	0.18602	0.24727	0.40940	0.36098	0.54290
4	20	0.15153	0.17858	0.42421	0.30078	0.43652
5	13	0.16743	0.29848	0.36342	0.25944	0.43290
5	30	0.15010	0.24475	0.32660	0.21132	0.40168

### The biochemical reliability of PCCF

In general, the patterns revealed by the clusters under different measures roughly agreed with each other. For instance, data set 5 had been grouped into 30 clusters by TransChisq and PoissonC measure, and these studies used five mouse photoreceptor and thirty-four cell-specific genes to demonstrate TransChisq and PoissonC measure were more efficient for analyzing SAGE data than PCC and Euclidean distance [7, 17]. The gene expression pattern of five photoreceptor genes showed high tag counts in late retinal development(adult), and thirty-four tags showed the most dynamic and cell-specific expression in the mouse neonatal retina(developmental stages *P*_0_ − *P*_6_) [7]. For comparison, we used PCCF and PCCP to group these 1,467 tags into 30 clusters also.

For these five rhodopsin tags, only PCCF was able to group them together, while other measures divided them into two clusters(Table 2). Moreover, these thirty-four ‘cell-specific’ tags were used to test the sensitivity and specificity of these measures. The comparison statistics of ‘cell-specific’ tags were summarized in Table 2. Here, for each of the different measures, its three most dynamic clusters that contained ‘cell-specific’ tags were selected. In Table 2, clusters of PCCF, TransChisq and PoissonC had no significant difference in these cell-specific genes. That is, PCCF was appropriate and reliable for analyzing SAGE data also.

**Table 2:** Statistics of 5 rhodopsin tags and 34 cell-specific genes.

5 rhodopsin genes	Measure	Numbers	Total	Sensitivity	Specificity
	PCCF	5	51	100%	9.80%
	PCCP	3	25	60%	12.0%
		2	16	40%	12.5%
	TransChisq	3	19	60%	15.8%
		2	10	40%	20%
	PoissonC	3	18	60%	16.7%
		2	17	40%	11.8%
34 Cell-specific genes	PCCF	11	37	32.4%	29.7%
		6	49	17.7%	12.2%
		5	48	14.7%	10.4%
	PCCP	6	22	17.6%	27.3%
		5	38	14.7%	13.2%
		6	58	17.6%	10.3%
	TransChisq	11	48	32.4%	22.9%
		4	36	11.8%	11.1%
		2	18	5.9%	11.1%
	PoissonC	10	45	29.4%	22.2%
		3	17	8.8%	17.6%
		2	24	5.9%	8.3%

The numbers in the third column were the numbers of rhodopsin genes(or cell-specific genes) in a cluster; total, the total number of cluster members; sensitivity, Numbers/5(or 34); specificity, Numbers/Total.

### The projecting reliability of PCA-F

The cumulative variance of principal components were commonly used to assess the projecting reliability of PCA [18]. Here, for all data sets in this paper, their cumulative variances of PCA-F, PCA-P and PCA-N were summarized in Table 3, where PCA-P and PCA-N are PCA of P-points and normalized points, respectively. For any data set, Table 3 showed that the cumulative variance of PCA-F and PCA-P had no significant difference, and PCA-P was slightly greater than PCA-F. Importantly, the cumulative variances of PCA-F and PCA-P were greater than 85% for any data set. However, for any data set, the cumulative variance of PCA-N was far less than PCA-F and PCA-P and only the data set 4 was slightly greater than 85%.

**Table 3:** The cumulative variances of PCA-F and PCA-N.

	Data	Var of the first PC	Var of the second PC	Cumulative variances
PCA-F	1	87.087%	8.379%	95.466%
PCA-P	1	91.394%	5.390%	96.784%
PCA-N	1	64.701%	12.499%	77.200%
PCA-F	2	66.624%	23.132%	89.756%
PCA-P	2	77.904%	14.005%	91.909%
PCA-N	2	37.410%	23.540%	60.950%
PCA-F	3	87.662%	5.030%	92.692%
PCA-P	3	89.625%	3.354%	92.979%
PCA-N	3	45.649%	11.261%	56.910%
PCA-F	4	95.305%	3.434%	98.739%
PCA-P	4	96.704%	2.151%	98.855%
PCA-N	4	84.136%	5.548%	89.684%
PCA-F	5	66.623%	18.654%	85.277%
PCA-P	5	74.742%	11.426%	86.168%
PCA-N	5	27.313%	15.473%	42.786%

Var of the first PC: the variance of the first principal components; Var of the second PC: the variance of the second principal components.

**Fig 1:**
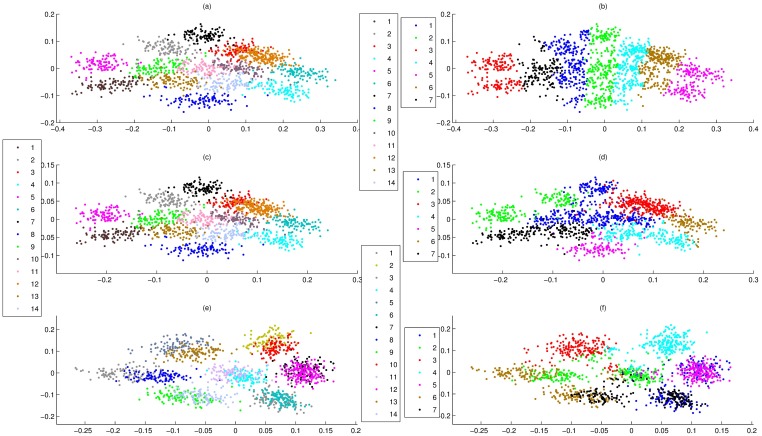
Overlay of clusters of data set 1 onto PCA-F, PCA-P and PCA-N maps, where data points were colored according to cluster membership. **(a)** PCA-F map of 14 populations. **(b)** Overlay of 7 clusters of PCCF onto PCA-F map. **(c)** PCA-P map of 14 populations. **(d)** Overlay of 7 clusters of PCCP onto PCA-P map. **(e)** PCA-N map of 14 populations. **(f)** Overlay of 7 clusters of PCC onto PCA-N map.

Furthermore, we used data set 1 to assess the statistical reliability of PCA-F. Here, according to population membership of points, data set 1 was mapped on PCA-F, PCA-P and PCA-N (Fig 1), respectively. From Fig 1(a) and 1(c), although there was little intermixing within adjacent populations, PCA-F and PCA-P were able to project most points of the same populations together. Importantly, even if all elements of points were relatively equivalent, PCA-F and PCA-P was able to project them together. For instance, PCA-F and PCA-P projected most points of (N(20,2),N(20,2),N(20,2),N(20,2)) together, where these points were marked by 11 in Fig 1(a) and 1(c). Moreover, PCA-N clearly projected points onto seven regions, but each of regions contained projections of two or more populations that had significant intermixing (Fig 1(e)).

### The feature of F-points

**Fig 2:**
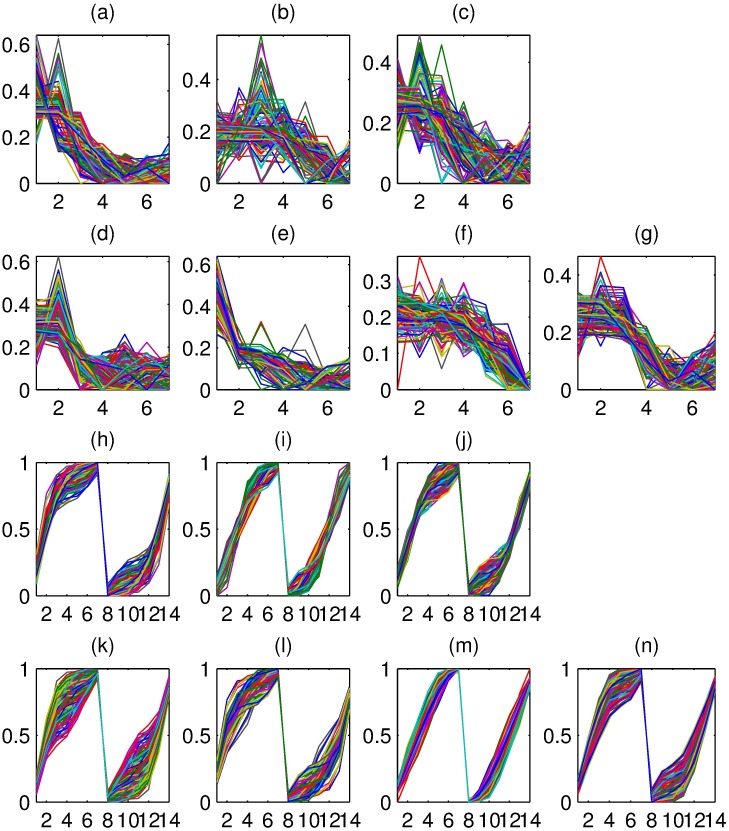
The profile plots of the normalized points and F-points. The *X*-axis represents the different time points. The *Y*-axis represents the expression level. **(a, b and c)** The profiles of normalized plots of three clusters of PCCF. **(d, e, f and g)** The profile of normalized plots of four clusters of PCC. **(h, i and j)** The F-points profile plots of three clusters of PCCF. **(k, l, m and n)** The F-points profile plots of four clusters of PCC.

Here, the down-regulate genes of data set 2 were selected to explore the feature of F-points, where data set 2 were divided into 12 clusters by PCCF and PCC, respectively. Moreover, these 3 clusters of PCCF and 4 clusters of PCC that contained down-regulate genes were selected, and the curve shape of F-points and normalized points of these clusters were shown in Fig 2. For clusters of PCCF, Fig 2 showed that the curve shape of F-points within any cluster were almost like capital *N*. But for F-points of different clusters that generated from PCCF, their elements had significant difference.

Furthermore, Fig 2 showed that the similarity between F-points and normalized points had significant difference. For instance, for genes in the second cluster of PCCF, the curve shape of their normalized points were with no specific patterns (Fig 2(b)), but there were only small differences for their F-points (Fig 2(i)).

### The consistency between PCA-F and PCCF

**Fig 3:**
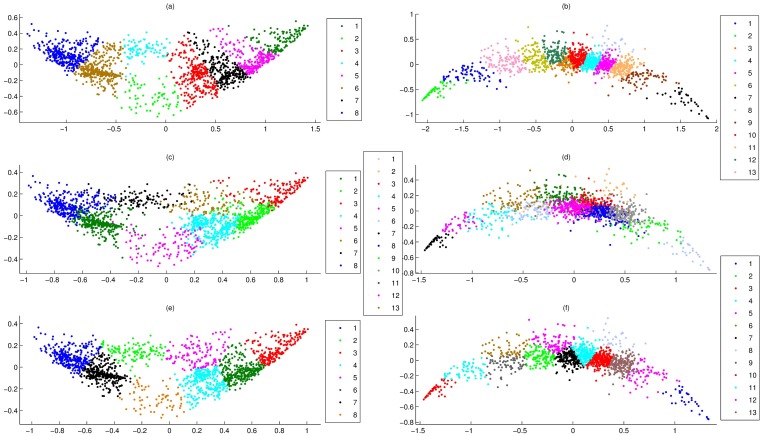
Overlay of clusters of data set 2 and 5 onto PCA-F and PCA-P maps. **(a)** Overlay of 8 clusters of PCCF of data set 2 onto PCA-F map. **(b)** Overlay of 13 clusters of PCCF of data set 5 onto PCA-F map. **(c)** Overlay of 8 clusters of PCCP of data set 2 onto PCA-P map. **(d)** Overlay of 13 clusters of PCCP of data set 5 onto PCA-P map. **(e)** Overlay of 8 clusters of EuF of data set 2 onto PCA-P map. **(f)** Overlay of 13 clusters of EuF of data set 5 onto PCA-P map.

When we use a measure to define the similarity of genes, a good visualization was that it was able to project similar points into the same regions. This was able to visually display by 2D maps of clustering results. Here, for data set 1, 2 and 5, their clusters of PCCF, PCCP and PCC were shown on PCA-F, PCA-P and PCA-N maps, where the clustering numbers of data set 1, 2 and 5 were 7, 8 and 13, respectively. Results showed that PCA-F gave a good visualization for any clustering result of PCCF (Figs 1(b), 3(a) and 3(b)), PCA-P maps had significant intermixing for any clustering result of PCCP (Figs 1(d), 3(c) and 3(d)), and PCA-N gave poor visualizations for clusters of PCC (Fig 1(f)). In fact, for clusters of PCCF, PCA-F was able to give a good visualization even if the clustering number was not very appropriate. For instance, for clusters of data set 1 that generated by PCCF, PCA-FO gave clear cluster boundary for clustering number from 2 to 12. These results clearly demonstrate that PCA-F was able to project similar points into the same regions.

Moreover, for a good visualization, its close projections should come from the similar points, and the feature could be evaluated by *S*2-value. Here, for each data set in this paper, its normalized points were divided into clusters by Euclidean, PCC, PCCP, EuF and PCCF, simultaneously. Then, *S*2-values of these clustering results were summarized in Table 4. For *S*2-value of any data, Table 4 showed that clusters of PCCF were the largest, and far more than other measures. That is, for projections of PCA-F, if they were close neighbours in 2D space, their corresponding F-points were Pearson correlation also.

**Table 4:** The *S*2-values of Eu, PCC, PCCP, EuF and PCCF.

Data	Clustering number	Euclidean	PCC	PCCP	EuF	PCCF
1	14	0.20427	0.18071	0.17269	0.37355	0.41520
1	7	0.33561	0.42601	0.28892	0.36729	0.44093
2	8	0.21844	0.12117	0.33842	0.34472	0.47512
2	12	0.18822	0.02316	0.27094	0.32635	0.41805
3	3	0.25896	0.22037	0.35470	0.35810	0.54903
3	7	0.17817	0.00127	0.12349	0.22670	0.37853
4	6	0.36049	-0.15155	0.53979	0.47601	0.70383
4	10	0.18378	-0.18443	0.37631	0.36038	0.59199
4	20	0.14921	-0.21595	0.29810	0.28821	0.40441
5	13	0.07048	-0.07371	0.14172	0.24359	0.37795
5	30	0.01168	-0.15203	0.02501	0.17687	0.19856

### Comparison of PCA-FO and PCA-F

**Fig 4:**
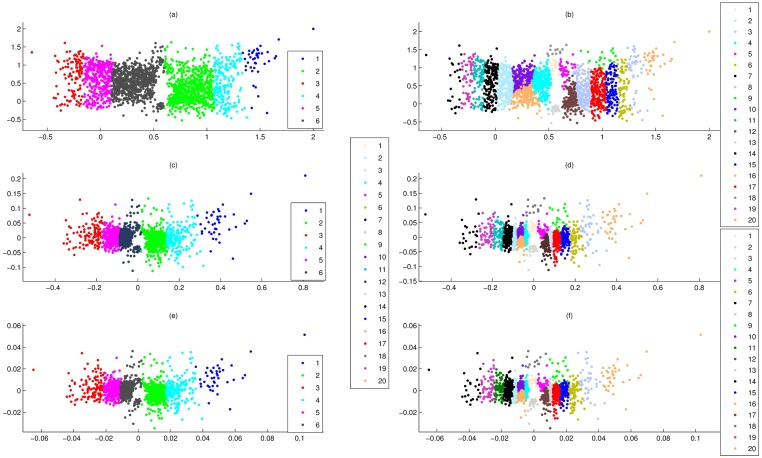
Overlay of clusters of data set 4 onto PCA-FO, PCA-F and PCA-N maps, where clusters were generated by PCCF. **(a)** Overlay of 6 clusters onto PCA-FO map. **(b)** Overlay of 20 clusters onto PCA-FO map. **(c)** Overlay of 6 clusters onto PCA-F map. **(d)** Overlay of 20 clusters onto PCA-F map. **(e)** Overlay of 6 clusters onto PCA-N map. **(f)** Overlay of 20 clusters onto PCA-N map.

Here, data set 4 were divided into 6 and 20 clusters by the PCCF, and these clustering results were overlaid on PCA-FO and PCA-F maps (Fig 4), respectively. Fig 4 showed that PCA-FO and PCA-F gave the good visualizations for any clustering result. However, for projections in the internal regions, PCA-F maps were crowded (Fig 4(c) and 4(d)), while PCA-FO maps were relatively loose and clear (Fig 4(a) and 4(b)).

In fact, for any of components of two nearest projections of PCA-FO, their spacing was greater than *l*/2*m*, where *l* was the largest exhibition size, *m* was the gene number of data set. In a limited display space, the feature of PCA-FO would assure that projections were relatively loose and clear. Furthermore, compared to PCA-F and PCA-FO, the position relationship of their projections were the same almost. In fact, for the first(or second) components of PCA-FO, their order of size were the same as PCA-F.

### Comparison of PCA-FO and t-SNE

Here, we also used the simple t-SNE to construct 2D projections of F-points, where we named t-SNE of F-points as t-SNE-F, and the dimension of the F-points was used as the perplexity value of t-SNE-F.

**Fig 5:**
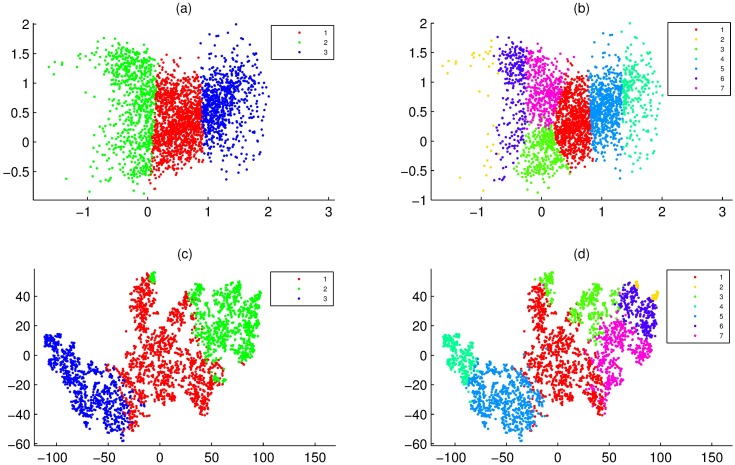
Overlay of clusters of data set 3 onto PCA-FO and t-SNE-F maps, where clusters were generated by PCCF. **(a)** Overlay of 3 clusters onto PCA-FO map. **(b)** Overlay of 7 clusters onto PCA-FO map. **(c)** Overlay of 3 clusters onto t-SNE-F map. **(d)** Overlay of 7 clusters onto t-SNE-F map.

Here, data set 3 was firstly divided into 3 and 7 clusters by PCCF, and then these clustering results were overlaid on PCA-FO and t-SNE-F maps (Fig 5). Fig 5(a) and 5(b) showed that PCA-FO gave these clustering results good 2D projections. However, Fig 5(c) and 5(d) showed that t-SNE-F maps had significant intermixing for any clustering result.

### The local and global validity of PCA-FO

**Fig 6:**
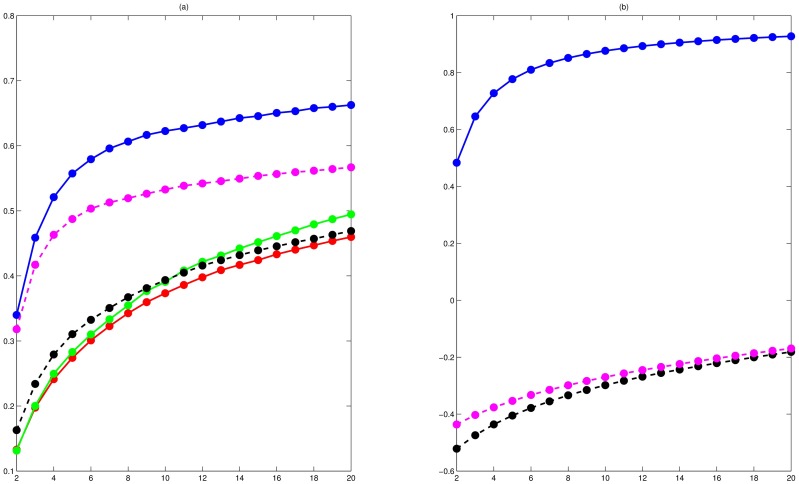
D-plot of data set 2. D-plot of PCA-FO, PCA-F, PCA-N t-SNE-F and t-SNE-N were displayed by green line, red line, gray dotted line, blue line and pink dotted line, respectively. **(a)** *D*_1_-plot of PCA-FO, PCA-F, PCA-N t-SNE-F and t-SNE-N. **(b)** *D*_2_-plot of PCA-FO, PCA-F, PCA-N t-SNE-F and t-SNE-N.

Here, *D*_1_-plot and *D*_2_-plot were used to assess the local and global validity of different dimension reduction techniques, where *D*_1_-plot and *D*_2_-plot of data set 2 were overlaid on Fig 6(a) and 6(b), respectively. For the local validity of PCA-FO, PCA-F and PCA-N, Fig 6(a) showed that they had no significant difference, but they were less than t-SNE-F and t-SNE-N. But for the global validity, PCA-FO, PCA-F and t-SNE-F were almost the same, and they were far better than t-SNE-N and PCA-N.

The poor global validity of t-SNE-N and PCA-N was able to explain that they gave the poor visualization for clusters of PCC. That is, the relationship of distantly normalized genes was not accurately depicted by t-SNE-N and PCA-N. But for t-SNE-F, its global validity was the same as PCA-FO, and its local validity was superior to PCA-FO. However, for clusters of PCCF, t-SNE-F maps had significant intermixing within adjacent clusters (Fig 5(c) and 5(d)). In fact, for these gene neighbors keep away from any clustering center, t-SNE-F tried to project them together, but PCCF did not necessarily group them together.

### The gene neighbor map of PCA-FO

**Fig 7:**
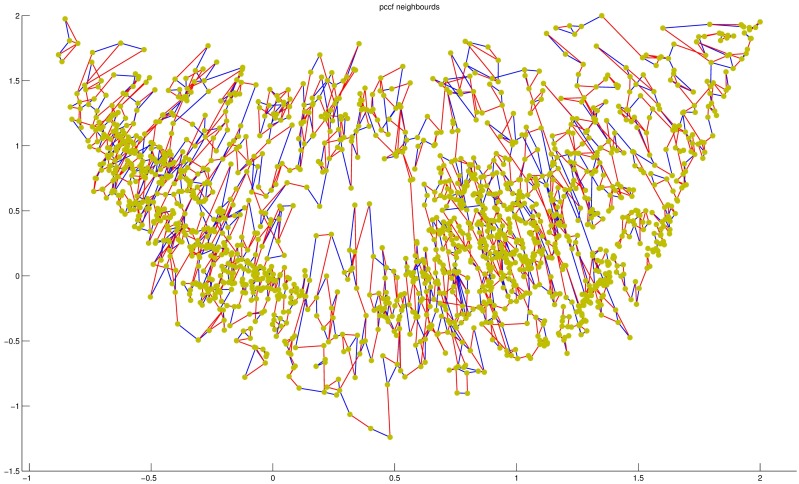
The gene neighbors of data set 2. The nearest and second closest neighbors of genes of data set 2, where the nearest gene neighbor were lined by red line, and second-closest gene neighbor were lined by blue line.

To readily see which nearby 2D points were truly similar, the nearest and second closest gene neighbor map was generated by PCA-FO. Here, we constructed the nearest and second closest gene neighbor map of data set 2, where the map was showed on Fig 7. Fig 7 showed that the majority of high dimension nearest neighbours were located close to one another in PCA-FO maps.

The gene neighbor map revealed the pairs of high dimensional points that were truly close, and which pairs were in fact distant in 2D space. Moreover, PCA-FO maps combined with nearest neighbour maps provided an intuitive means to understand the relationship between clusters and the affiliation of genes with specific clusters.

## Discussion

For the modified cumulative probability, although they are the one-to-one mapping with their normalized points, their magnitude has significant differences, which can result in PCA-P to give the poor visualizations for clusters of PCCP. Moreover, for the different position elements of a normalized point, their superposed opportunity are not consistent in the modified cumulative probability, which can make PCCP excessively dependent on the first few elements of normalized points. Here, the defect of the modified cumulative probability is removed by F-points. That is, the magnitude of F-points is the same, and F-points assure that the superposed opportunity of all elements of normalized points are consistent. Importantly, for data set 2 and 4, PCA-N gave good visualizations for clusters of PCCF also (such as Fig 4(e) and 4(f)). That is, F-points retain the difference of the normalized genes.

For a complex gene expression data set, a difficult issue in K-means is the estimation of *K*, the number of clusters. If *K* is unknown, starting with arbitrary random *K* is a relatively poor method. Here, the defect of K-means are partially weakened by PCCF and PCA-F. That is, for the similar expression behavior genes, even if the number of clusters is not very appropriate, PCCF can group them into appropriate clusters, and PCA-F is able to reveal their relationships also.

## Conclusion

In this paper, we clearly demonstrate that PCCF is more reliable for analyzing gene expression data compared to other commonly used measures. Moreover, for clusters of PCCF, PCA-F give them good visualizations. The success of PCCF and PCA-F indicates that the effective methods for analyzing large-scale gene expression data must be based on an understanding of the biological nature of the experimental data.

## Supporting information

S1 File A freely available MATLAB code that can obtain F-points, PCA-FO and nearest neighbour maps for a data set. (ZIP)

S2 File Data set 2. (XLS)

S3 File Data set 3. (XLS)

S4 File Data set 4. (XLS)

S5 File Data set 5. (XLS)
